# Supplementary material for: Convergence and Divergence of Common and Rare Variants of Autism Spectrum Disorders in Tissue-specific Pathways and Gene Networks
Source: Res Sq. 2025 May 15:rs.3.rs-6581159. Preprint. [Version 1] doi: 10.21203/rs.3.rs-6581159/v1 (PMC12136192; doi:10.21203/rs.3.rs-6581159/v1)
Supplement: 1 [file NIHPPRS6581159V1-supplement-1.pdf]

SUPPLEMENTARY FIGURE LEGENDS

Supplementary Figure 1: Brain tissue key driver subnetworks demonstrating convergence of rare and common variants: SCN8A, AMPH, ATP9A.
Key driver subnetworks **A)** SCN8A (sodium voltage-gated channel alpha subunit 8), **B)** AMPH (amphiphysin), and **C)** ATP9A (ATPase phospholipid transporting 9A) were identified within the brain Bayesian network and showed high enrichment of both rare and common ASD-associated variants. These key drivers and their first-degree network neighbors were visualized using Cytoscape and colored based on their SFARI database ASD confidence level stratification or by their ASD GWAS common variant disease association strength. Uncolored nodes were genes within the Bayesian network but were not found to be associated with either grouping. EnrichR was used to analyze the key driver subnetwork genes to generate pathway annotation terms, with the top annotations displayed below each network. Terms were ranked based on their *-log10* p-value.

Supplementary Figure 2: Peripheral tissue key driver subnetworks demonstrating high enrichment of common variants or a convergence of rare and common variants: PRR36 and PCHD7.
Key driver subnetworks **A)** PRR36 (proline rich 36), and **B)** PCDH7 (protocadherin 7) were identified from the endocrine and digestive Bayesian networks, respectively. PRR36 displayed high enrichment of common variants while PCDH7 showed a convergence of both rare and common variants. These key drivers and their first-degree network neighbors were visualized using Cytoscape and colored based on their SFARI database ASD confidence level stratification or by their ASD GWAS common variant disease association strength. Uncolored nodes were genes within the Bayesian network but were not found to be associated with either grouping. EnrichR was used to analyze the key driver subnetwork genes to generate pathway annotation terms, with the top annotations displayed below each network. Terms were ranked based on their *-log10* p-value.

TABLES

Supplementary Table 1. Summary of eQTLs and sQTLs from the Genotype Tissue Expression Portal (GTEx) database for Mergeomics analysis.
eQTLs and sQTLs from 14 brain regions and 35 peripheral tissues were retrieved from the GTEx database to be used for tissue-specific mapping of SNPs from the ASD GWAS to genes that are potentially regulated by the SNPs in each tissue. Four tissues did not have eQTL/sQTL data (bladder, endocervix, fallopian tube, renal medulla), so distance-based mapping was utilized for their respective tissues during Marker Set Enrichment Analysis.

Region	Tissue	eQTL Count	sQTL Count
Peripheral	Adipose (Subcutaneous)	1501725	649559
Peripheral	Adipose (Visceral Omentum)	1116992	501342
Peripheral	Adrenal Gland	606549	266580
Brain	Amygdala	241511	84934
Brain	Anterior Cingulate Cortex (Brodmann Area 24)	360811	121320
Peripheral	Artery (Aorta)	1137522	446515
Peripheral	Artery (Coronary)	468104	231587
Peripheral	Artery (Tibial)	1528215	615729
Peripheral	Breast Mammary Tissue	915137	478320
Brain	Caudate	592299	183573
Brain	Cerebellar Hemisphere	689639	234153
Brain	Cerebellum	838323	276313
Peripheral	Colon (Sigmoid)	861273	367653
Peripheral	Colon (Transverse)	946365	392568
Brain	Cortex	675220	208046
Peripheral	Cultured Fibroblasts	1558412	604866
Peripheral	EBV-transformed Lymphocytes	303559	215630
Peripheral	Esophagus (Gastroesophageal Junction)	891705	375284
Peripheral	Esophagus (Mucosa)	1374993	480793
Peripheral	Esophagus (Muscularis)	1344979	499704
Brain	Frontal Cortex (Brodmann Area 9)	511541	168139
Peripheral	Heart (Atrial Appendage)	962118	360824
Peripheral	Heart (Left Ventricle)	850330	286330
Brain	Hippocampus	374601	128103
Brain	Hypothalamus	385593	147721
Peripheral	Kidney Cortex	76350	35421
Peripheral	Liver	407126	158972
Peripheral	Lung	1261501	567609
Peripheral	Minor Salivary Gland	294404	143965
Peripheral	Muscle (Skeletal)	1405527	574674
Peripheral	Nerve (Tibial)	1720751	659871
Brain	Nucleus Accumbens	580998	199042
Peripheral	Ovary	370124	197505
Peripheral	Pancreas	818024	251970
Brain	Pituitary	713991	326113

Peripheral	Prostate	514066	258156
Brain	Putamen	477659	137298
Peripheral	Skin (Non-Sun-Exposed Suprapubic)	1409730	564897
Peripheral	Skin (Sun-Exposed Lower Leg)	1632271	638497
Peripheral	Small Intestine (Terminal Ileum)	414021	195522
Brain	Spinal Cord (Cervical C1)	293087	111631
Peripheral	Spleen	770698	279622
Peripheral	Stomach	706670	295700
Brain	Substantia Nigra	198962	75770
Peripheral	Testis	1554886	954055
Peripheral	Thyroid	1765762	682833
Peripheral	Uterus	216551	138022
Peripheral	Vagina	224692	139644
Peripheral	Whole Blood	1276546	377472

875

Supplementary Table 2: Summary of tissue-specific Bayesian networks constructed using GTEx transcriptome data.

Bayesian networks were constructed for individual tissues and further merged into seven Bayesian networks representing major biological systems to reduce sparsity.

Network Name	Individual Tissues	Node Number	Edge Number
Adipose	Adipose (Subcutaneous), Adipose (Visceral Omentum)	2262	17758
Brain	Amygdala, Anterior Cingulate Cortex, Caudate, Cerebellar Hemisphere, Cortex, Frontal Cortex, Hippocampus, Hypothalamus, Nucleus Accumbens, Putamen, Pituitary, Spinal Cord, Substantia Nigra	37866	223949
Cardiovascular	Artery (Aorta), Artery (Coronary), Artery (Tibial), Heart (Atrial Appendage), Heart (Left Ventricle)	11241	41168
Digestive	Colon (Sigmoid), Colon (Transverse), Esophagus (Gastroesophageal Junction), Esophagus (Mucosa), Esophagus (Muscularis), Liver, Minor Salivary Gland, Pancreas, Small Intestine (Terminal Ileum), Stomach	14259	68401
Endocrine	Adrenal, Ovary, Pituitary, Testis, Thyroid	14720	37690
Female Reproductive	Breast Mammary Tissue, Endocervix, Ectocervix, Fallopian Tube, Ovary, Uterus, Vagina	10020	25117
Immune	EBV Lymphocytes, Spleen, Whole Blood	10430	21815
Immune-Adipose	Adipose (Subcutaneous), Adipose (Visceral Omentum), EBV Lymphocytes, Spleen, Whole Blood	12692	39573

Supplementary Table 3: Top 10 coexpression modules enriched for ASD GWAS signals from Marker Set Enrichment Analysis (MSEA).
The top ten gene sets from MSEA were ranked based on false discovery rate (FDR) of ASD GWAS enrichment. Tissue corresponds to the tissue origin of the gene coexpression modules from GTEx data; module name indicates which coexpression module within a given tissue showed ASD GWAS enrichment. The top annotations indicate the pathway terms that had the highest enrichment p-values based on pathway annotation of the genes in each tissue-specific coexpression module. The top informative genes were selected based on the SNP p-values from the ASD GWAS, prioritizing those with previous evidence of association with ASD, including known coding genes and others with established roles in ASD pathogenesis or pathophysiology.

	Tissue	Module	FDR	Top Annotations	Top Informative Genes in GWAS
**1**	Anterior Cingulate Cortex	blue	1.27E-24	Protein Degradation, Long Term Potentiation, Immune System	*AF131216.5, ARL17A, CTC-498M16.4, GABBR1, KANSL1, LRRC37A, MAPK8IP1P2, MAPT, PLEKHM1, TMEM161B-AS1*
**2**	Amygdala	black	3.07E-24	Spliceosome Activity, Proteasome Activity, Electron Transport	*ARL17A, CRHR1, GABBR1, KANSL1, LINC02210, LRRC37A, LRRC37A2, MAPK8IP1P2, MAPT, PLEKHM1*
**3**	Liver	turquoise	5.59E-23	Cell Cycle Regulation, DNA Repair, mTORC1 Activity	*ARL17A, DND1P1, KANSL1, KANSL1-AS1, LINC02210, LRRC37A2, MAPK8IP1P2, MAPT-IT1, PLEKHM1, RP11-259G18.3*
**4**	EBV Lymphocytes	blue	2.01E-19	TNF-α Signaling, Pancreatic Cancer, Neurotrophin Signaling	*ARHGAP27, BLK, DND1P1, FAM167A, HLA-A, KANSL1, LINC02210, MAPK8IP1P2, RP11-259G18.3, RP11-707O23.1*
**5**	Minor Salivary Gland	light-green	6.69E-19	RNA Splicing, TGF-β signaling, Membrane Trafficking	*ARL17A, FAM85B, KANSL1, KANSL1-AS1, LINC02210, LRRC37A2, LRRC37A4P, MAPK8IP1P2, RP11-259G18.1, RP11-259G18.3*
**6**	Frontal Cortex	gray60	7.77E-19	Immune Response and Regulation, Neuronal, Synaptic Processes	*ARL17A, CRHR1, GABBR1, KANSL1, KIZ, LINC02210, LRRC37A, MAPT, PLEKHM1, TDH*
**7**	Uterus	turquoise	3.33E-16	Transcription, mRNA processing	*ARL17A, DND1P1, KANSL1, KANSL1-AS1, LINC02210, LRRC37A, LRRC37A2, MAPK8IP1P1, RP11-259G18.3, RP11-707O23.1*
**8**	Cerebellum	purple	1.04E-15	Energy Metabolism, Cellular Stress Response, Oxygen Transport	*ARL17A, CRHR1, FMNL1, GABBR1, KANSL1, LINC02210, MAPT, NSF, PLEKHM1, TDH*
**9**	Cortex	blue	2.49E-15	Olfactory Signal Transduction	*ARHGAP27, CRHR1, DND1P1, GABBR1, KANSL1, LINC02210, LRRC37A, MAPT, PLEKHM1, RP11-259G18.1*
**10**	Testis	magenta	2.52E-13	Cell Cycle Regulation, DNA Repair	*LINC00208, MACROD2, MSRA, PINX1, PTBP2, RP11-177H2.2, RP11-227D2.3, RP11-6N13.1, TDH, XRN2*

Supplementary Table 4: Top network key drivers in brain and peripheral tissues with subnetwork enrichment for both rare variants (RV) and common variants (CV) of ASD at an enrichment p-value < 0.05.

KD	RV Score	CV Score	Tissue	Tissue Region
**ADD2**	2.07E-09	8.12E-07	Substantia Nigra, Pituitary, Frontal Cortex	Brain
**AMPH**	1.43E-04	4.63E-03	Substantia Nigra	Brain
**ATP9A**	8.83E-06	6.23E-04	Cerebellum	Brain
**CALD1**	2.22E-03	2.49E-02	Colon Sigmoid	Digestive
**CD74**	3.31E-02	5.32E-04	Colon Sigmoid	Digestive
**DYNC1H1**	1.17E-02	7.85E-03	Cerebellum	Brain
**DYNC1I1**	3.01E-02	1.17E-02	Cerebellum	Brain
**MAP2K1**	1.91E-04	3.12E-02	Caudate	Brain
**PCDH7**	1.61E-05	3.66E-02	Colon Sigmoid	Digestive
**PLPP3**	6E-04	3.69E-02	Putamen, Frontal Cortex	Brain
**RBFOX2**	2.42E-05	1.39E-02	Substantia Nigra	Brain
**SCN8A**	5.99E-04	6.5E-04	Substantia Nigra	Brain
**SNAP91**	9.16E-08	1.68E-02	Substantia Nigra	Brain
**SYP**	7.96E-04	2.18E-02	Substantia Nigra, Pituitary	Brain
**TMEM130**	3.45E-08	1.24E-03	Pituitary	Brain
**XKR4**	2.13E-02	6.79E-03	Colon Sigmoid	Digestive
